# Chemo-photothermal therapy combination elicits anti-tumor immunity against advanced metastatic cancer

**DOI:** 10.1038/s41467-018-03473-9

**Published:** 2018-03-14

**Authors:** Jutaek Nam, Sejin Son, Lukasz J. Ochyl, Rui Kuai, Anna Schwendeman, James J. Moon

**Affiliations:** 1 Department of Pharmaceutical Sciences, University of Michigan, Ann Arbor, MI 48109 USA; 2 Biointerfaces Institute, University of Michigan, Ann Arbor, MI 48109 USA; 3 Department of Biomedical Engineering, University of Michigan, Ann Arbor, MI 48109 USA

## Abstract

Photothermal therapy (PTT) is a promising cancer treatment modality, but PTT generally requires direct access to the source of light irradiation, thus precluding its utility against disseminated, metastatic tumors. Here, we demonstrate that PTT combined with chemotherapy can trigger potent anti-tumor immunity against disseminated tumors. Specifically, we have developed polydopamine-coated spiky gold nanoparticles as a new photothermal agent with extensive photothermal stability and efficiency. Strikingly, a single round of PTT combined with a sub-therapeutic dose of doxorubicin can elicit robust anti-tumor immune responses and eliminate local as well as untreated, distant tumors in >85% of animals bearing CT26 colon carcinoma. We also demonstrate their therapeutic efficacy against TC-1 submucosa-lung metastasis, a highly aggressive model for advanced head and neck squamous cell carcinoma (HNSCC). Our study sheds new light on a previously unrecognized, immunological facet of chemo-photothermal therapy and may lead to new therapeutic strategies against advanced cancer.

## Abstract (web summary)

Photothermal therapy (PTT) for cancer treatment is currently limited to local, accessible, tumors. Here the authors show that PTT combination with chemotherapy, by stimulating an immune response, is effective against distant tumors and establishes immune memory, thus providing a strategy to target metastatic disease.

## Introduction

There is an urgent demand for effective cancer therapies that can eliminate large solid tumors as well as disseminated, metastatic nodules, while simultaneously preventing tumor recurrence. Thermal ablation of tumor cells with photothermal therapy (PTT) is a promising approach for the treatment of local tumors^1,2^. By local administration of photosensitizers and minimally invasive near-infrared (NIR) radiation, hyperthermia induced by PTT can be controlled to minimize the damage to non-targeted tissues^3^. Yet, it is difficult to completely eradicate large tumors with conventional PTT due to residual tumor mass at the treatment margins^2^. While combination strategies have been widely reported to improve the overall efficacy^4–9^, their main site of action is restricted to local tumors, and it remains impractical to use PTT against disseminated, metastatic tumors that are inaccessible to the source of NIR. Intriguingly, recent studies have shown that hyperthermia can induce dying tumor cells to release antigens, pro-inflammatory cytokines, and immunogenic intracellular substrates, thus promoting immune activation^10–12^. Nevertheless, prior studies have mainly employed models with a single primary tumor, often in immunocompromised mice, in order to assess direct killing of tumor cells by PTT^1,3,6–9^. Thus, the overall contribution of immune stimulation on anti-tumor efficacy of PTT remains unclear, especially in the light of recent reports documenting PTT-mediated immunosuppression within the tumor microenvironment^13^.

Gold nanoparticles (GNPs) are biocompatible photosensitizers that exhibit strong surface plasmon resonance (SPR) and efficient conversion of light to heat^14^. NIR-absorbing GNPs typically require anisotropic morphology and/or rough surface, as in the case of spiky gold nanoparticles (SGNPs) with large NIR absorption cross-section and high photothermal efficiency^15^. However, anisotropic nano-spikes of SGNPs are thermodynamically unstable and vulnerable to photothermal reshaping to low surface energy structures^16–21^. As there is an inverse relationship between hyperthermia and tumor relapse^22^, quick loss of the structure-directed NIR-responsiveness of SGNPs limits their in vivo applications^23,24^. While surface passivation layers have been reported to alleviate photothermal deformation in vitro^18–21^, their in vivo photothermal stability, anti-tumor efficacy, as well as their impact on the tumor microenvironment remain unknown.

**Fig. 1:**
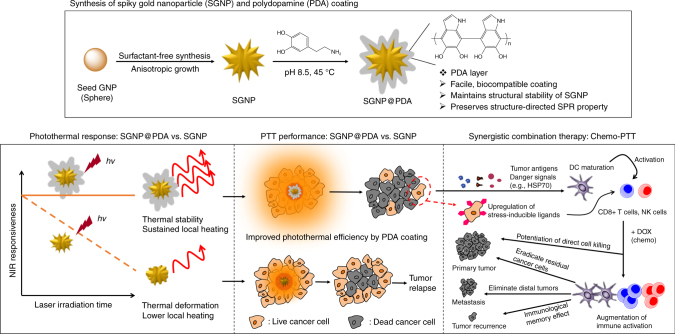
The schematic illustration shows the development of spiky gold nanoparticles (SGNPs) coated with PDA (SGNP@PDA) as a new photothermal agent with extensive photothermal stability and efficiency. The combination chemo-photothermal therapy triggered potent anti-tumor immunity in vivo and exerted strong anti-tumor efficacy against local primary tumors and untreated, distal tumors, while simultaneously establishing long-term immunity against tumor recurrence

Here, we have developed a simple and versatile strategy to produce a photothermally stable, highly efficient NIR photothermal agent based on SGNPs (Fig. 1). We demonstrate that polydopamine (PDA) coating, previously used for various biological applications such as drug delivery and biologic sensing^9,25–27^, confers robust photothermal stability to nano-spike structures of SGNPs and significantly improves their photothermal efficiency in vitro and in vivo. Importantly, we show that chemo-photothermal therapy (chemo-PTT), based on PDA-coated SGNPs and a sub-therapeutic dose of doxorubicin (DOX), elicits robust anti-tumor responses in both cellular (CD8+ T and NK cells) and humoral compartments. Chemo-PTT eliminates residual tumor cells from locally treated tumors and exerts an abscopal effect against untreated, distant tumors, leading to a remarkable survival rate of >85% in a bilateral murine tumor model of CT26 colon carcinoma. Furthermore, treated animals exhibit long-term resistance against tumor re-challenge, indicating establishment of immunological memory against tumor recurrence. Chemo-PTT also exerts strong anti-tumor efficacy in a highly aggressive model of TC-1 submucosa-lung metastasis—a pre-clinical model of advanced head and neck squamous cell carcinoma (HNSCC) that closely mimics the clinical evaluations of PTT with silica–gold nanoshells (AuroLase®)^28^. Overall, our study demonstrates previously unappreciated immunological aspects of chemo-PTT and may offer a new platform for the next-generation cancer therapy.

## Results

### Synthesis and characterization of SGNPs and PDA-coated SGNPs

**Fig. 2:**
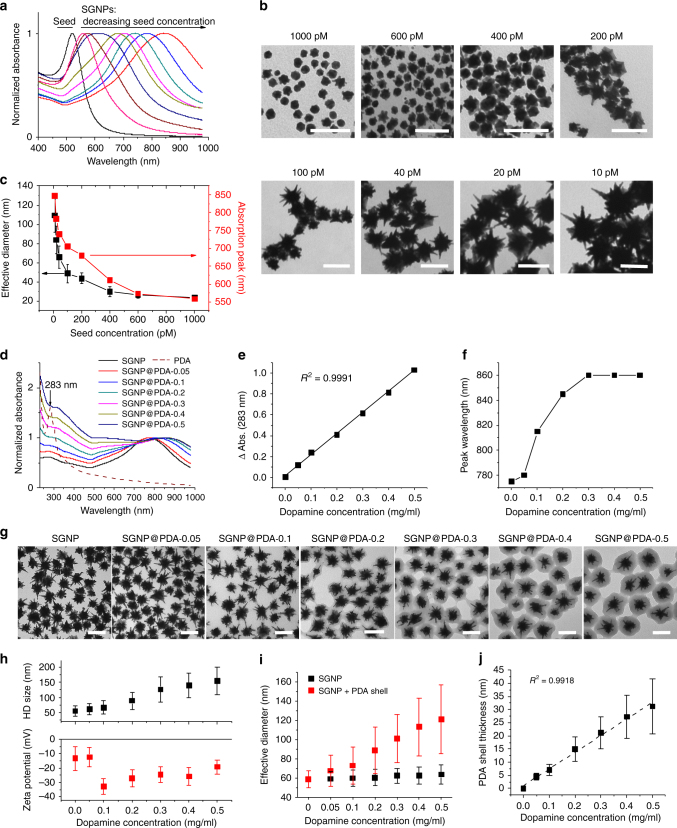
Synthesis and characterization of SGNPs and SGNP@PDAs. **a**–**c** Shown are the absorption spectra (**a**), TEM images (**b**), and correlation between the concentration of seed NPs and either the effective diameter or absorption peak wavelength (**c**) of SGNPs. **d**–**f** Shown are the absorption spectra (**d**), changes in 283 nm absorbance (**e**), and shift in the peak wavelength (**f**) of SGNP@PDAs depending on the concentration of dopamine. SGNP with 775 nm absorption peak was prepared on a large scale and employed for PDA coating study. The number followed by PDA denotes the concentration of dopamine (mg/ml). **g**, **h** TEM images of SGNP@PDAs (**g**), and their hydrodynamic size and zeta potential (**h**). **i**, **j** Effective diameters of SGNP with or without the PDA shell (**i**) and thickness of the PDA shell (**j**) determined by analyzing the projected areas of NPs in **g**. Scale bars are 100 nm. The data show mean ± s.d., representative from 2–3 independent experiments. More than 100 particles were counted for TEM analyses shown in **c**, **i**, and **j**

We synthesized citrate-stabilized GNPs^29^ as the seed for SGNP synthesis. Seed GNPs exhibited their peak absorbance at 520 nm and a spherical morphology with 16.1±2.2 nm diameter (Supplementary Figure 1). We grew nano-spikes on the seed GNPs by a surfactant-free, seed-mediated growth method, employing AgNO_3_ and ascorbic acid as the structure-directing and reducing agents, respectively^30^. The resulting SGNPs were surface-passivated with poly(ethylene glycol) (PEG) methyl ether thiol to improve their colloidal stability. We were able to readily tune the opto-physical properties of SGNPs; decreasing the input concentration of the seed GNPs resulted in gradual red-shift in the absorbance spectra of SGNPs (Fig. 2a) and increased the particle size and length of the nano-spikes (Fig. 2b). Their effective diameter, calculated from the projected areas of individual particles in transmission electron microscope (TEM) images, was directly correlated with their absorption red-shift (Fig. 2c). For SGNPs with >50 nm diameter, their SPR extended to the NIR region (Fig. 2a, c), which is attributable to the high aspect ratio of nano-spikes^30^. In contrast, spherical GNPs in the 100 nm size range generally exhibit an absorbance peak at <600 nm^31^.

Based on their NIR characteristics, we chose SGNPs with the absorption peak at 775 nm for coating with PDA, using spontaneous polymerization of dopamine on SGNPs in a mild basic condition^32^. As dopamine concentration increased from 0 to 0.5 mg/ml, the resulting nanoparticles, termed SGNP@PDA, exhibited an increased absorbance peak at 283 nm (ascribed to PDA; Fig. 2d, e) as well as distinct red-shift of the NIR absorption peak, reaching its plateau at 860 nm with 0.3 mg/ml dopamine, which reflected changes in local dielectric environment (Fig. 2d, f)^33^. TEM images showed homogeneous SGNPs with distinct PDA shell, whose thickness increased with the increasing dopamine concentration (Fig. 2g, Supplementary Figure 2). Accordingly, the hydrodynamic size of the particles gradually increased from 55 ± 18 nm for bare SGNPs to 150 ± 45 nm for SGNP@PDA-0.5, while their zeta potential decreased to the −35 mV to −20 mV range (Fig. 2h), probably due to deprotonation of the phenolic groups on the PDA shell^34^. Notably, we did not detect any self-polymerized PDA particles without SGNP cores, probably due to the low dopamine concentration. While the effective diameter of the SGNP core remained unchanged, the total effective diameter of particles (SGNP core plus PDA shell) grew linearly with the increasing dopamine concentration (Fig. 2i). The thickness of the PDA shell was finely tuned from 5 nm to 30 nm, depending on the input dopamine concentration (Fig. 2j). Overall, we have successfully synthesized NIR-absorbing, PDA-coated SGNPs with tunable opto-physical properties.

### Extensive photothermal stability of PDA-coated SGNPs

**Fig. 3:**
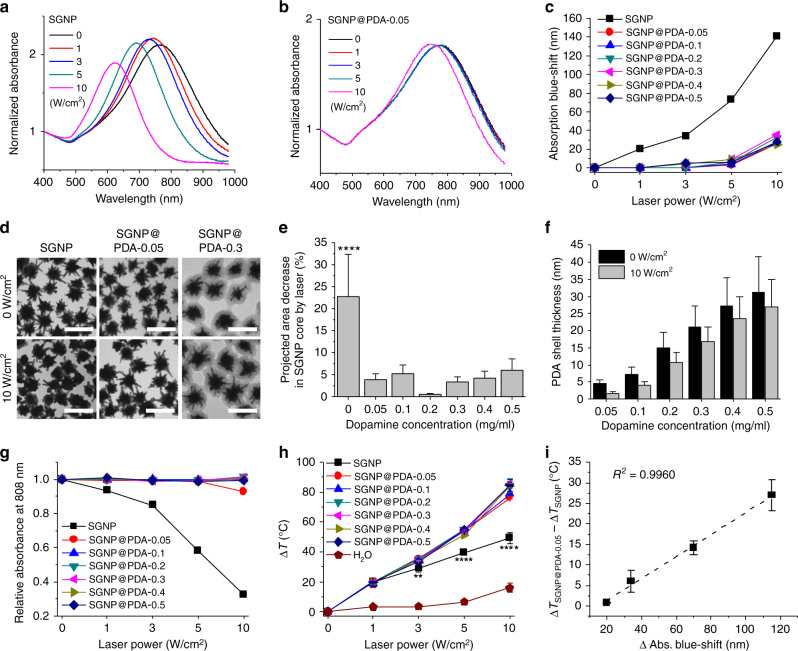
Improved photothermal efficiency of SGNP@PDA. **a**, **b** Absorption spectra of SGNP (**a**) and SGNP@PDA-0.05 (**b**) after 808 nm laser irradiation at 0 (no irradiation), 1, 3, 5, 10 W/cm^2^ for 30 min. **c** Blue-shift of the absorption peak wavelength for SGNP and SGNP@PDAs after laser irradiation. **d** Representative TEM images of SGNP, SGNP@PDA-0.05, SGNP@PDA-0.3 before (0 W/cm^2^) and after 10 W/cm^2^ laser irradiation. Scale bars are 100 nm. **e**, **f** Quantitative analysis of TEM images for the decrease in the SGNP core area (**e**) and thickness of the PDA layer before (0 W/cm^2^) and after 10 W/cm^2^ laser irradiation (**f**). **g**, **h** Relative changes in 808 nm absorbance of SGNP and SGNP@PDAs (**g**) and the increase in temperature after laser irradiation at varying laser power (**h**). **i** Relationship between absorbance blue-shift and temperature increase calculated by subtracting the values of SGNP from that of SGNP@PDA-0.05. The data show mean ± s.d., representative from 2–3 independent experiments (*n* = 3). More than 100 particles were counted for TEM analysis shown in **e**, **f**. **P < 0.01, ****P < 0.0001, analyzed by one-way ANOVA (**e**) or two-way ANOVA (**h**) with Bonferroni multiple comparison post-test. Asterisks indicate statistically significant differences between SGNP vs. all other SGNP@PDA groups

Next, we examined the impact of PDA coating on the NIR-responsiveness of SGNPs. Bare SGNPs exhibited extensive blue-shift of their absorption peak after 30 min exposure to 808 nm continuous-wave laser with a power density as low as 1 W/cm^2^ (Fig. 3a). In stark contrast, all SGNP@PDA formulations tested, including ~5 nm PDA shell (SGNP@PDA-0.05), largely retained their absorption spectra (Fig. 3b, Supplementary Figure 3). Quantitatively, after exposure to 10 W/cm^2^, bare SGNPs exhibited ~140 nm absorption blue-shift and drastic deformation of nano-spikes into a thermodynamically more stable spherical form^16–21^ (Fig. 3c–e). In sharp contrast, SGNP@PDAs at all PDA concentrations tested displayed minimal absorption blue-shift and retained their nano-spikes, albeit a slight reduction (3–4 nm) in PDA thickness was noted post 30 min laser irradiation at 10 W/cm^2^ (Fig. 3c–f, Supplementary Figure 4).

Accordingly, SGNP@PDAs maintained absorbance at 808 nm (abbreviated as Abs808) at laser power densities of 1–10 W/cm^2^ (Fig. 3g) with strong photothermal heating capability (Fig. 3 h, Supplementary Figure 5). In contrast, bare SGNPs exhibited a progressive decrease in Abs808 and produced significantly lower temperature increase (Δ*T*) (Fig. 3g, h). The degree of absorption blue-shift was well-correlated to Δ*T* between SGNP and SGNP@PDA-0.05 (Fig. 3i), showing the impact of the PDA coating on the preservation of Abs808 and photothermal heating efficiency. Notably, the PDA layer also improved the long-term stability of SGNP@PDAs, as shown by the minimal absorption blue-shift after 30 days of storage at 4 °C in PBS, whereas bare SGNPs exhibited prominent absorption blue-shift (Supplementary Figure 6). Overall, these results demonstrate that PDA coating on SGNPs prevented deformation of nano-spikes and improved their photothermal efficacy by sustaining NIR-responsiveness. Based on their physicochemical properties and photothermal stability, we used the SGNP@PDA-0.1 formulation for all the subsequent studies.

### PTT with SGNP@PDA exerts anti-tumor efficacy and immunity

**Fig. 4:**
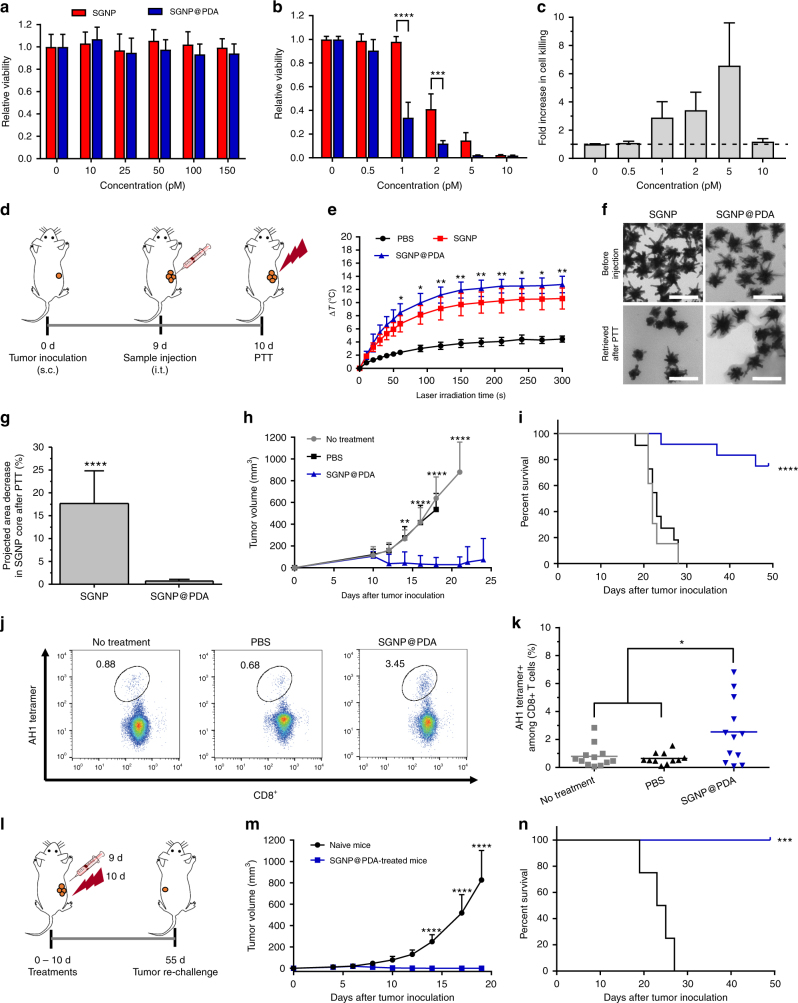
Potent therapeutic efficacy and anti-tumor T-cell responses achieved by PTT with SGNP@PDA. **a**, **b** Viability of CT26 colon carcinoma cells in vitro after treatment with varying concentrations of SGNP or SGNP@PDA, followed by 24 h incubation in dark (**a**) or laser irradiation at 10 W/cm^2^ for 5 min and further 24 h incubation (**b**). **c** Fold increase in cell killing by SGNP@PDA compared with SGNP. **d**–**g** Shown are the schematic illustration of the PTT regimen (**d**), temperature increase in tumors during laser irradiation at 1 W/cm^2^ for 5 min (**e**), TEM images of SGNP and SGNP@PDA before injection or after retrieval from tumors in vivo post PTT (**f**), and quantitative analysis of a decrease in SGNP core area from TEM images in **f** (**g**). **h**, **i** Tumor growth (**h**) and Kaplan–Meier survival curve (**i**) of CT26 tumor-bearing mice without any treatment or after laser irradiation post administration of PBS or 100 fmol of SGNP@PDA. **j**, **k** Representative scatter plots (**j**) and frequency (**k**) of AH1-specific CD8+ T cells in peripheral blood mononuclear cells, measured by flow cytometry after 7 days post laser irradiation. **l**–**n** Schematic of the PTT regimen (**l**), tumor growth (**m**), and Kaplan–Meier survival curve (**n**) of animals for the tumor re-challenge study. The data show mean ± s.d., representative from 2–3 independent experiments; *n* = 3 (**a**, **b**), *n* = 5 (**e**–**g**), *n* = 11–13 (**h**–**n**). *P < 0.05, **P < 0.01, ***P < 0.001, and ****P < 0.0001, analyzed by two-way ANOVA (**b**, **e**, **h**, **m**) or one-way ANOVA (**k**) with Bonferroni multiple comparisons post-test; unpaired two-tailed *t*-test (**g**); or log-rank (Mantel–Cox) test (**i**, **n**). Asterisks indicate statistically significant differences between SGNP@PDA vs. SGNP (**e**); between SGNP@PDA vs. no treatment (**h**); and between SGNP@PDA vs. all other groups (**i**)

We next examined SGNP@PDAs as a PTT agent using the CT26 colorectal carcinoma model. Both SGNP and SGNP@PDA were biocompatible and non-toxic at all concentrations tested when incubated with CT26 cells in vitro (Fig. 4a). Upon laser irradiation (10 W/cm^2^ for 5 min), SGNP@PDAs killed CT26 cells more efficiently than SGNPs (3–7 fold increase for 1–5 pM; Fig. 4b, c). To confirm the results in vivo, we inoculated BALB/c mice subcutaneously on the right flank with 5 × 10^5^ CT26 cells. On day 9 when tumors were ~100 mm^3^, we performed intratumoral injection of 50 fmol SGNP@PDAs or SGNPs, followed by laser irradiation (808 nm, 1 W/cm^2^ for 5 min) 1 day later (Fig. 4d). PTT with SGNP@PDA increased the intratumoral temperature by +13 °C (Fig. 4e) and efficiently inhibited tumor growth, with 40% of animals eliminating tumors (Supplementary Figure 7). In contrast, animals treated with bare SGNPs exhibited a significantly lower temperature increase of +10 °C (Fig. 4e) and rapid tumor re-growth within 1 week of PTT, with 100% of animals moribund by day 45 (Supplementary Figure 7). Interestingly, TEM analysis of nanoparticles recovered from tumors revealed that laser irradiation ablated nano-spiky branches on bare SGNPs in vivo and reduced their projected area by 18%, whereas nano-spikes and PDA shells remained intact in SGNP@PDAs (Fig. 4f, g). Overall, the protective PDA layer improved the photothermal efficiency and in vivo performance of SGNPs per a given particle dose and laser fluence by preventing their photothermal reshaping and loss of NIR-responsiveness. Based on these evidence as well as potential dose-sparing and reduced safety concerns, we have focused the subsequent studies on SGNP@PDA.

Next, we sought to understand the impact of PTT with SGNP@PDA on anti-tumor T-cell immunity, as local hyperthermia of tumors has been shown to trigger immune responses^2,10,11,13^. BALB/c mice were inoculated with CT26 cells and treated with 100 fmol of SGNP@PDA (2-fold higher dose than Fig. 4d). PTT with SGNP@PDA resulted in tumor regression, with 75% of animals eradicating tumors and remaining tumor-free up to 50 days (Fig. 4h, i). In contrast, mice without any treatment or treated with PBS plus laser irradiation succumbed to tumor growth within 30 days. We next investigated the impact of PTT on induction of anti-tumor CD8+ T-cell responses by analyzing peripheral blood mononuclear cells (PBMCs) on day 7 after laser irradiation for the frequency of CD8+ T cells specific to the immune-dominant AH1 epitope (SPSYVYHQF), using AH1 peptide-major histocompatibility complex class I (H-2L^d^) tetramer^35^. Mice that received PTT with SGNP@PDA elicited markedly enhanced CD8+ T-cell responses, generating ~4-fold higher frequency of AH1-specific CD8+ T cells than the PBS control group (Fig. 4j, k). Furthermore, we evaluated the animals for long-term anti-tumor immunity by re-challenging survivors from Fig. 4i with subcutaneous injection of 5 × 10^5^ CT26 cells on the contralateral flank on day 55 (Fig. 4l). All animals previously treated with SGNP@PDA-PTT rejected CT26 tumor cells, whereas all naive mice succumbed to CT26 tumor within 30 days (Fig. 4m, n). Overall, these results indicate that PTT with SGNP@PDA elicited robust anti-tumor T-cell immunity and exerted strong therapeutic efficacy against large (~100 mm^3^) primary tumors and tumor recurrence.

### Chemo-PTT for elimination of local and distal tumors

**Fig. 5:**
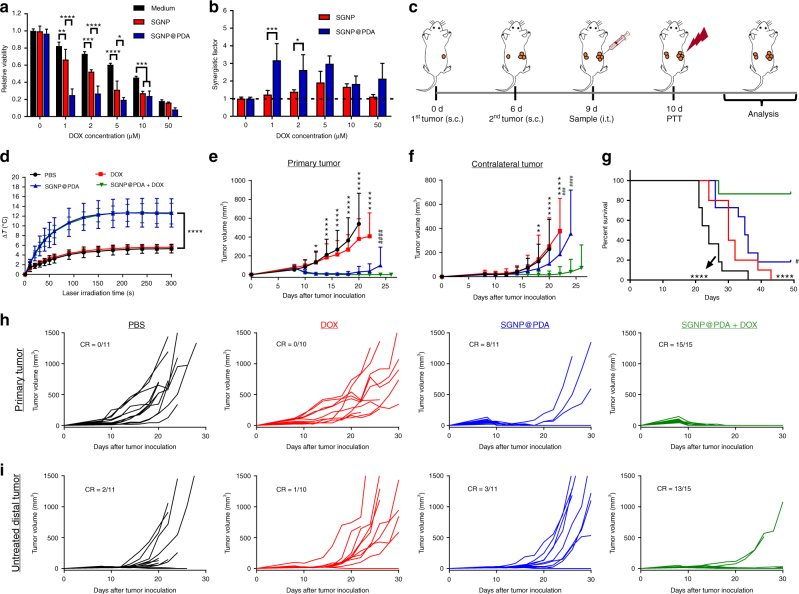
Combination chemo-photothermal therapy exerts potent anti-tumor efficacy against local and disseminated tumors. **a** Viability of CT26 cells after co-treatment of varying concentrations of DOX with 0.5 pM of SGNP or SGNP@PDA followed by laser irradiation at 10 W/cm^2^ for 5 min and further 24 h incubation. “Medium” indicates DOX treatment alone. **b** Synergistic factor of combination therapy calculated based on the viability in **a**. **c**, **d** Schematic for the combination therapy in a bilateral tumor model (**c**), temperature increase in the primary tumor during 5 min laser irradiation at 1 W/cm^2^ (**d**). **e**–**i** Shown are the average tumor growth (**e**, **f**) and individual tumor growth (**h**, **i**) of the treated primary tumors (**e**, **h**) and untreated contralateral tumors (**f**, **i**) with fraction of complete tumor regression (CR), and the overall Kaplan–Meier survival curves (**g**). The data show mean ± s.d. from a representative experiment from 2–3 independent experiments; *n* = 3 (**a**, **b**), *n* = 5 (**d**), *n* = 10–15 (**e**–**i**). *P < 0.05, ***P < 0.001, and ****P < 0.0001, analyzed by two-way ANOVA (**a**, **b**, **d**, **e**, **f**, **g**), followed by Bonferroni multiple comparisons post-test; or log-rank (Mantel–Cox) test (**g**); * in **d**, **e**, **f**, **g** indicates statistically significant differences between SGNP@PDA + DOX vs. PBS or DOX; # in **e**, **f**, **g** indicates statistically significant differences between SGNP@PDA + DOX vs. SGNP@PDA

Many groups have reported the synergy between PTT and DOX-based chemotherapy for treatment of local tumors^5–9^; however, prior studies have used either human tumor xenograft models or single tumor models^6–9^. As DOX has been shown to trigger immune responses^36,37^, we next sought to delineate the impact of SGNP@PDA-mediated PTT combined with DOX on anti-tumor immunity and its efficacy against disseminated tumors. With the addition of DOX, PTT with SGNP@PDA killed CT26 cells more effectively than PTT with SGNP in vitro (Fig. 5a), with strong synergy for the combination of SGNP@PDA plus DOX (Fig. 5b). SGNP@PDA generated efficient local heating over the threshold for chemo-potentiation, while SGNP required a higher dose to achieve a similar synergistic effect (Supplementary Figure 8). In the absence of the particles or laser irradiation, tumor cell killing was reduced, suggesting a crucial role played by each component (Supplementary Figures 9, 10).

To examine the therapeutic potential of the chemo-PTT combination in vivo, we employed a dual tumor model (Fig. 5c), where only one flank tumor was treated with chemo-PTT, while the contralateral tumor was monitored without any treatment in order to examine for systemic anti-tumor efficacy induced by chemo-PTT. This dual tumor model is very challenging for conventional PTT strategies, as they generally require direct access to the source of NIR for effective treatment. We inoculated BALB/c mice subcutaneously with 5 × 10^5^ CT26 cells on the right (primary tumor) and left (contralateral tumor) flank on day 0 and 6, respectively. On day 9, the primary tumor was administered with 100 fmol SGNP@PDA and/or 1.36 mg/kg DOX—a sub-therapeutic dose of DOX as a mono-chemotherapy^5–9^. On day 10, the primary tumors in all animals received laser irradiation (1 W/cm^2^ for 5 min), whereas the contralateral tumors were left untreated to assess the impact of systemic anti-tumor immunity. Laser irradiation of SGNP@PDA groups with or without DOX resulted in similar temperature profiles in tumors (Fig. 5d). At this low dose regimen, free DOX treatment had a negligible impact on tumor growth (Fig. 5e–i). Importantly, SGNP@PDA + DOX treatment led to complete regression of primary tumors in 100% mice, whereas a subset of primary tumors treated with SGNP@PDA alone relapsed by day 20 (Fig. 5e, h). Remarkably, SGNP@PDA + DOX treatment also exhibited strong anti-tumor efficacy in the untreated, contralateral tumors, with tumor regression observed in 13 out of 15 animals (Fig. 5f, i). Overall, SGNP@PDA + DOX treatment led to complete regression of both primary and contralateral tumors and a long-term survival for 87% of animals (Fig. 5g–i), compared with 0% and 18% survival rates for DOX and SGNP@PDA, respectively (Fig. 5g). Moreover, all survivors in the SGNP@PDA + DOX group also rejected tumor re-challenge performed on day 55 with CT26 cells, indicating establishment of immunological memory against tumor recurrence (Supplementary Figure 11).

### Immunological impact of chemo-PTT

**Fig. 6:**
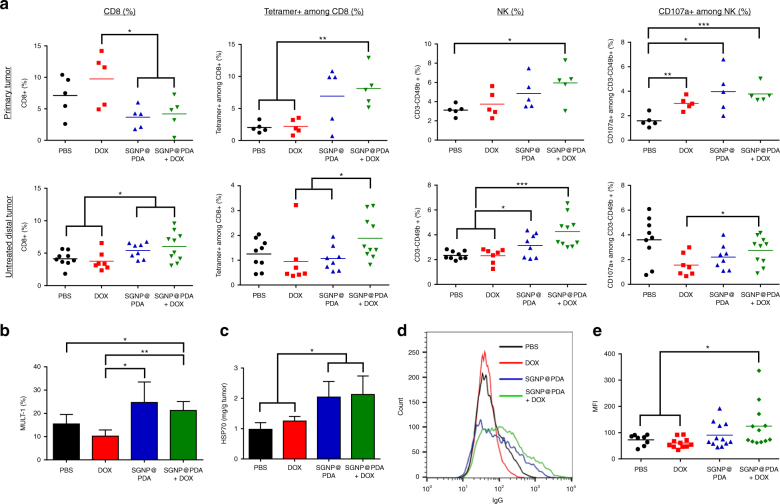
Systemic and local anti-tumor immunity triggered by chemo-photothermal therapy. Bilateral tumor model was established using CT26 cells and treated as in Fig. 5c. **a** Frequencies of CD8+ T cells, AH1-specific CD8+ T cells, NK cells, and CD107a+ NK cells were measured among tumor-infiltrating lymphocytes in primary and contralateral tumors on day 17 (7 days post PTT). **b** Percent of MULT-1-positive cells in the tumor was analyzed by flow cytometry. **c** Intratumoral concentration of HSP70 was measured by ELISA. **d**, **e** Representative histogram plot (**d**) and mean fluorescence intensity (MFI) (**e**) of CT26 cell-binding sera IgG collected and analyzed after 20 days of PTT. The data show mean ± s.d., representative from 2–3 independent experiments; *n* = 5–10 (**a**–**c**), *n* = 8–12 (**e**); *P < 0.05, **P < 0.01, and ***P < 0.001, analyzed by one-away ANOVA, followed by Bonferroni multiple comparisons post-test

We next sought to understand how SGNP@PDA + DOX treatment exerted systemic anti-tumor effect. Regression of untreated, distal tumors mediated by SGNP@PDA + DOX treatment shown above (Fig. 5f, i) was not due to direct chemotherapeutic effect of DOX, as the sub-therapeutic dose of DOX used in our studies did not impact the primary tumor growth nor accumulate in the contralateral tumors (Supplementary Figure 12). We analyzed the impact of chemo-PTT on the innate and adaptive immune compartments on day 17 using the same bilateral tumor model as in Fig. 5c. After laser irradiation, SGNP@PDA + DOX treatment triggered upregulation of MHC-II and CD40 among dendritic cells (DCs) in tumor-draining lymph nodes (Supplementary Figure 13) and elicited robust AH1-specific CD8+ T-cell responses in both primary and distal tumors (3.7-fold greater than DOX group in the primary tumor; and 2.0- and 1.8-fold greater than DOX group and SGNP@PDA group, respectively, in the contralateral tumor; Fig. 6a). Furthermore, chemo-PTT also increased the frequency of NK cells in the primary and distal tumors and induced their activation as shown by upregulated CD107a^38^, lysosomal-associated membrane protein-1 (Fig. 6a). PTT also promoted CT26 cancer cells to upregulate mouse UL16-binding protein-like transcript 1 (MULT-1), a stress-induced ligand for NKG2D receptor on NK cells^39^ (Fig. 6b) as well as HSP70, a key ‘danger’ signal involved in recruitment and activation of DCs and lymphocytes^40,41^ (Fig. 6c). Moreover, SGNP@PDA + DOX treatment induced serum IgG that bound live CT26 cells (Fig. 6d, e). Collectively, these results show that SGNP@PDA + DOX treatment potently activated distinct innate and adaptive immune compartments within the local and distal tumor microenvironment.

### Interrogation of immune compartments for anti-tumor efficacy

**Fig. 7:**
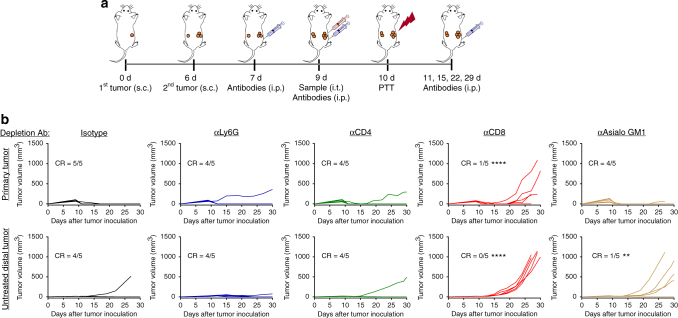
Anti-tumor efficacy of chemo-photothermal therapy mediated by different immune compartments. **a** Bilateral tumor model was established using CT26 cells, and tumor-bearing mice were intraperitoneally injected with depletion antibodies during and post PTT with SGNP@PDA + DOX. **b** Shown are individual tumor growth curves with the fraction of complete tumor regression (CR) for the mice treated with antibodies targeted against neutrophils (αLy6G), CD4+ T cells (αCD4), CD8+ T cells (αCD8), and NK cells (αAsialo GM1). Isotype antibody was used as a control group. **P < 0.01 and ****P < 0.0001, analyzed by two-way ANOVA with Bonferroni multiple comparisons post-test in comparison with isotype control. Asterisks indicate statistical differences from the isotype control group

Having shown tumor infiltration and activation of CD8+ T cells and NK cells mediated by SGNP@PDA + DOX treatment, we sought to delineate their roles in anti-tumor efficacy against local and distal tumors. We established the same bilateral tumor model as in Fig. 5c, performed PTT with SGNP@PDA + DOX, and administered isotype control antibody or depleting antibodies targeted against neutrophils (αLy6G), CD4+ T cells (αCD4), CD8+ T cells (αCD8), or NK cells (αAsialo GM1), starting on day 7 to ensure complete depletion of target cells throughout the experimental period (Fig. 7a). Administration of αLy6G or αCD4 IgG did not negate the therapeutic efficacy (Fig. 7b), suggesting that neutrophils and CD4+ T cells are not essential for SGNP@PDA + DOX treatment. In stark contrast, administration of αCD8 IgG led to relapse of primary tumors and quick growth of contralateral tumors (Fig. 7b). On the other hand, administration of αAsialo GM1 IgG abrogated the therapeutic efficacy of SGNP@PDA + DOX against contralateral tumors, but not against primary tumors (Fig. 7b). Collectively, these results show that CD8+ T cells are the main effector cells for eradication of distal tumors as well as prevention of relapse at the primary site, while NK cells cooperate with CD8+ T cells to eliminate distal tumors, thus highlighting their distinct roles in regression of primary and metastatic tumors.

### Chemo-PTT against advanced head and neck cancer

**Fig. 8:**
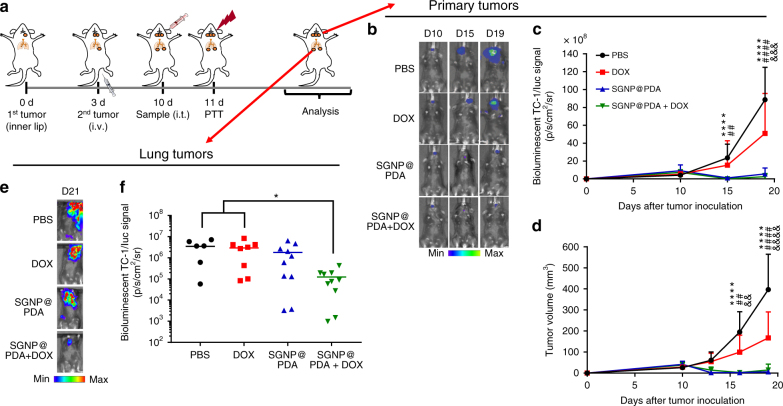
Therapeutic efficacy of chemo-photothermal therapy against advanced head and neck squamous cell carcinoma. **a** Schematic for the TC-1 submucosa-lung metastasis model and the treatment regimen. **b**–**d** Growth of TC-1/luc lip tumors were visualized using IVIS (**b**) and quantified by either the bioluminescence signal (**c**) or direct measurements of the tumor volume (**d**). **e** Representative in vivo bioluminescence images of TC-1/luc lung tumors visualized on day 21 with IVIS after shielding the primary lip tumors with a piece of black paper, and **f** the corresponding intensity of the bioluminescence signal. The data show mean ± s.d. with *n* = 6–10. *P < 0.05, **P < 0.01, ***P < 0.001, and ****P < 0.0001, analyzed by two-way ANOVA (**c**, **d**) or one-way ANOVA (**f**) with Bonferroni multiple comparisons post-test. *, #, & in **c**, **d** indicate statistically significant differences between PBS vs. SGNP@PDA (± DOX) (∗); DOX vs. SGNP@PDA (± DOX) (#); or PBS vs. DOX (&)

Finally, we sought to examine the therapeutic potential of our strategy in a more advanced tumor model. We employed highly aggressive TC-1 submucosa-lung tumors as a model for advanced HNSCC that frequently metastases to lungs^42^. This model also simulates the clinical evaluations of PTT with silica–gold nanoshells (AuroLase®) in the setting of advanced HNSCC^28^. C57BL/6 mice were inoculated at a submucosal site of the inner lip with firefly luciferase-expressing TC-1 cells (TC-1/luc)—a widely accepted, orthotopic model of HPV-associated HNSCC^43,44^. In addition, the animals were inoculated intravenously (i.v.) with TC-1/luc cells on day 3 to establish metastatic lung nodules (Fig. 8a). On day 10, when inner lip tumors reached ~50 mm^3^ in volume, we measured the bioluminescence signal with In Vivo Imaging System (IVIS) (Fig. 8b) and administered 100 fmol SGNP@PDA and/or 1.36 mg/kg DOX into the primary inner lip tumors. On the following day, the primary tumors were exposed to laser irradiation (1 W/cm^2^ for 5 min) and monitored over time.

Whereas DOX treatment alone had minimal effect on tumor growth, PTT with SGNP@PDA or SGNP@PDA + DOX effectively ablated the inner lip tumors as quantified by the bioluminescence signal that correlated well with the direct measurements of tumor volume (Fig. 8c, d). Importantly, bioluminescence imaging performed on day 21 revealed that SGNP@PDA + DOX treatment significantly inhibited the growth of TC-1/luc lung tumors, leading to a 28-fold and 24-fold decrease in the bioluminescence signal, compared with PBS and DOX treatment groups, respectively (Fig. 8e, f). SGNP@PDA + DOX treatment also resulted in a 14-fold decrease in the bioluminescence signal, compared with the SGNP@PDA monotherapy although this difference was not statistically significant due to high variations in this advanced tumor model. Nevertheless, these results show that the combination chemo-PTT treatment effectively ablates primary tumors while strongly inhibiting the growth of untreated, distal tumors.

## Discussion

SGNPs represent one of the most thermodynamically unstable structures because of their anisotropic morphology and high surface energy of nano-spike branches with large surface area. To address these limitations, surface passivation layers, such as alkanethiols and polymers, have been introduced for prolonged storage in vitro at room temperature or slightly elevated temperature^18–21^. However, their thermal stability at a temperature range relevant for PTT (>50 °C)^45^ is yet to be demonstrated. In this study, we have shown that PDA coating serves as a surface passivation layer that endows strong photothermal stability to SGNPs in extreme heating conditions (>90 °C). We speculate that such robust protection is due to inherent adhesive property of PDA that leads to a strong interaction with SGNP surface atoms through metal coordination or chelation^46^, thus limiting thermal diffusion and rearrangements of surface atoms and inhibiting subsequent shape reconstruction.

The combination of PTT and chemotherapy has been a subject of intense investigation^4–9^. While various chemotherapeutic agents, including DOX, have been shown to improve the therapeutic efficacy of PTT, their main mode of action has been attributed to direct cell killing^4–9^, and their effects on the local and systemic immune responses as well as viable approaches to exploit them for systemic cancer therapy remained unexplored. Here, we present, to the best of our knowledge, the first demonstration that a single round of PTT combined with a sub-therapeutic dose of DOX can markedly enhance elicitation of anti-tumor CD8+ T cells and NK cells, overcome immunosuppression in tumors, and exert systemic anti-tumor immunity that can effectively eliminate local as well as distant, untreated tumors in >85% of animals. Furthermore, all survivors were protected against secondary tumor re-challenge, highlighting the therapeutic potential of chemo-PTT against not only established tumors but also tumor relapse.

Interestingly, PTT as a monotherapy was effective at ablating primary tumors and preventing tumor engraftment upon tumor cell re-challenge (Fig. 4); however, PTT monotherapy failed to show any therapeutic efficacy in advanced, metastatic tumor models of CT26 and TC-1 (Figs. 5, 8) known for their immunosuppressive tumor microenvironment^47,48^. This is in line with prior studies that have suggested dampening of PTT-induced immune activation within the tumor microenvironment^13^. While recent studies have addressed immunosuppression by supplementing PTT with immunotherapy, those approaches employed defined adjuvant molecules^49^, adoptive T-cell transfer^13^, or repeated systemic administrations of immune checkpoint blockers^50,51^. In contrast, our strategy based on chemo-PTT allows in situ cancer vaccination, where PTT-mediated thermal damage of tumor cells in combination with immunogenic chemotherapy effectively prime the local tumor microenvironment for immune activation. Mechanistically, we have demonstrated that PTT with SGNP@PDA promoted upregulation of intratumoral HSP70 and MULT-1. HSP70 is known to recruit DCs, serve as a chaperone for antigen uptake by DCs, and promote cross-priming of T-cell responses^12,40,41^. HSP70 also enhances cytotoxic effector functions of NK cells via stress-inducible upregulation of MULT-1, a stimulatory ligand for NKG2D on NK cells^39,52^. Furthermore, DOX-based chemotherapy amplified the immune activation initiated by PTT. It is known that DOX induces immunogenic cell death, promotes activation of DCs and CD8+ T cells, and sensitizes tumor cells to killing by T cells and NK cells^36,37,53^. Consequently, chemo-PTT orchestrated intratumoral infiltration of CD8+ T cells and NK cells and their activation of effector functions. Our antibody depletion studies have shown that CD8+ T cells and NK cells are the main effector cells against CT26 tumors, which is in line with other reports^54,55^. Interestingly, our data have suggested that CD8+ T cells were necessary and sufficient for regression and prevention of tumor relapse in the local primary tumors, while regression of untreated, distant tumors required both CD8+ T cells and NK cells. We speculate that this difference stems from more efficient infiltration of antigen-specific CD8+ T cells into primary tumors (~8% and ~2% tetramer+ CD8+ T cells in the primary vs. contralateral tumors, respectively, Fig. 6a) and subsequent local T-cell activation induced by hyperthermia^56^ and DOX^36,37,53^. Notably, the dose of DOX used in our study was sub-therapeutic. Only when we combined SGNP@PDA and DOX therapies did we observe amplification of NK cells and CD8+ T cells that exerted strong anti-tumor efficacy against local as well as distant, untreated tumors. Although it is beyond the scope of our current study, the dosing and kinetics of DOX delivery should be further optimized to maximize the therapeutic efficacy, especially in the setting of advanced cancer.

In summary, we report a facile yet versatile PDA coating strategy that offers a new nano-platform with markedly improved photothermal stability and efficiency. Our studies show that chemo-PTT tuned to elicit anti-tumor immunity can exert striking therapeutic effects against primary and disseminated tumors. Our strategy for in situ cancer vaccination based on chemo-PTT may offer a new treatment modality for advanced cancer.

## Methods

### Reagents and instruments

L-ascorbic acid was obtained from Fisher Chemical. RPMI 1640, penicillin–streptomycin, and ACK lysis buffer were purchased from Gibco. Fetal bovine serum was obtained from Corning. The sources of antibodies for flow cytometric analysis were indicated where they appeared in the text. Other chemicals were obtained from Sigma-Aldrich, and all reagents were used as received. UV-Vis absorption spectra were obtained using BioTek synergy neo microplate reader. TEM images were acquired using JEOL 1400-plus and analyzed using the ImageJ software (NIH, Bethesda, MD). Hydrodynamic size and zeta potential were measured using Malvern Zetasizer Nano ZSP. Laser irradiation was performed using a 808-nm cw diode laser (China Daheng Group Inc., Beijing, China). Temperature increase was recorded using a mini-hypodermic thermocouple probe coupled with digital thermometer (OMEGA Engineering, Inc.). Flow cytometric analyses were performed using Cyan 5 (Beckman Coulter) and the data were analyzed using FlowJo 10.2 software.

### Synthesis of citrate-stabilized GNPs

Fresh stock of 1.5 mmol of HAuCl_4_ was dissolved in 300 ml deionized (DI) water and boiled to reflux for 30 min. Then, 4.5 mmol of sodium citrate tribasic dehydrate was quickly added with vigorous stirring. The solution color changed from yellow to red within 5 min as gold ion was reduced to form GNPs. The mixture was boiled for 10 min and then cooled for 30 min at room temperature. The resulting citrate-stabilized GNPs were stored at 4 °C until further use.

### Synthesis of SGNPs

SGNPs were prepared using seed-mediated, surfactant-free method as described in the literature with slight modifications^30^. SGNPs with a series of opto-physical properties were synthesized by controlling the amount of seed citrate-stabilized GNPs. Specifically, 50–5000 μl of the above citrate-stabilized GNPs were diluted to 20 ml with DI water and sequentially mixed with 4 μmol of HAuCl_4_, 20 μl of 1 M HCl, 600 nmol of AgNO_3_ with vigorous stirring. After further stirring for 1 min, 8 μmol of L-ascorbic acid was added, which induced abrupt color change from red to greenish black in 10 s indicating the SGNP formation. Finally, 200 nmol of poly(ethylene glycol) methyl ether thiol (MW 6000) was added to quench the reaction and stabilize the SGNPs. The mixture was stirred for another 2 h at room temperature and centrifuged at 3000×*g* for 60 min to remove excess unreacted reagents. For PDA coating study, SGNPs were prepared on a larger scale with the peak absorbance at ~775 nm. Briefly, 10 ml of citrate-stabilized seed GNPs were diluted in 300 ml DI water and 60 μmol of HAuCl_4_, 300 μl of 1 M HCl, 9 μmol of AgNO_3_, 120 μmol of L-ascorbic acid, and 3 μmol of poly(ethylene glycol) methyl ether thiol were added in a similar manner and allowed to react for another 2 h. After centrifugation, the pellet was re-dispersed in 1 ml DI water and passed through illustra NAP-10 column (GE healthcare life sciences) for further purification. The resulting SGNPs were stored at 4 °C until further use.

### PDA coating on SGNPs

PDA coating was conducted using spontaneous polymerization of dopamine in a mild alkaline condition^32^. The thickness of PDA was controlled by the concentration of dopamine in the reaction solution. We found mild heating is required to obtain stable and substantially thick PDA coating on SGNPs probably due to chemically inert PEG modification and high surface energy of nano-spike. Specifically, 2 pmol of SGNPs were diluted in 20 ml of 10 mM Tris buffer (pH 8.5) and added with 0.05–0.5 mg/ml dopamine as indicated in the result section. After stirring for 30 min at 45 °C, 200 nmol of poly(ethylene glycol) methyl ether thiol was mixed and reacted for another 30 min to quench the reaction and stabilize the particles. The resulting PDA-coated SGNPs (SGNP@PDA) were centrifuged at 3000×*g* for 60 min to remove excess unreacted reagents. The pellets were re-dispersed in 1 ml of 10 mM Tris buffer and further reacted with 1 μmol of poly(ethylene glycol) methyl ether thiol for 2 h at room temperature. Finally, the mixtures were centrifuged at 3000×*g* for 10 min and the pellets were re-dispersed in DI water and stored at 4 °C. The endotoxin level for each in vivo dose of 100 fmol SGNP@PDA was measured to be less than 0.05 EU/dose, as determined by the LAL Chromogenic Endotoxin Quantitation Kit (Pierce).

### Photothermal response of SGNP and SGNP@PDAs

SGNP and SGNP@PDAs were diluted in DI water with the sample concentration matched to have an extinction of 1 at the excitation wavelength. The laser light was introduced to sample solutions in a glass cuvette while temperature was measured by inserting a thermocouple probe in the middle of solution. The equilibrium temperature was reached within 10–15 min. Samples were recovered after laser irradiation for 30 min, and their opto-physical changes were analyzed by absorption spectra and TEM images.

### In vitro cell experiments

CT26 murine colorectal cancer cells were obtained from the American Type Culture Collection (Rockville, MD), and maintained in RPMI 1640 supplemented 10% fetal bovine serum and 1% penicillin–streptomycin. Throughout the studies, all cells were used as received and tested negative for mycoplasma contamination and rodent pathogens. CT26 cells were plated at a density of 1 × 10^4^ cells/well in 96-well plates and incubated overnight at 37 °C under 5% CO_2_. To determine the cytotoxicity of SGNP and SGNP@PDA, the cells were incubated with 0, 10, 25, 50, 100, and 150 pM of SGNP or SGNP@PDA for 24 h. After removing free particles by rinsing with cell culture medium, Cell Counting Kit-8 solution (CCK-8, Dojindo Laboratories, Kumamoto, Japan) was added to each well of the plate according to manufacturer’s instruction and absorbance at 450 nm was measured using a microplate reader after 2 h incubation. Relative viability was calculated as the ratio of the absorbance to the non-sample treated cells (0 pM). For the evaluation of photothermal therapeutic efficiency, the cells were added with SGNP or SGNP@PDA at their concentrations of 0, 0.5, 1, 2, 5, and 10 pM and immediately exposed to laser irradiation for 5 min (10 W/cm^2^). The viability was measured using CCK-8 after further 24 h incubation, and the results are expressed as a relative viability to the non-sample treated, laser-irradiated cells (0 pM). To investigate the synergistic effect of chemo-photothermal combination cancer therapy, SGNP or SGNP@PDA (0.5 pM or 1 pM) were added to the cells either alone or with DOX (1, 2, 5, 10, 50 µM), followed by immediate laser irradiation for 5 min (10 W/cm^2^). Cells were further incubated for 24 h, and viability was measured using CCK-8 and expressed relative to the non-sample treated, laser-irradiated cells. For a quantitative analysis, synergistic factors were calculated by dividing the predicted additive viability by the viability of combination therapy^6^. Synergistic factor >1 indicates synergistically enhanced cell killing by combination when compared to sequential treatment of the individual modality.

### In vivo cancer therapy

Animals were cared for following federal, state, and local guidelines. All work performed on animals was in accordance with and approved by University Committee on Use and Care of Animals (UCUCA) at University of Michigan, Ann Arbor. Female BALB/c or C57BL/6 (5–6 weeks) were purchased from Envigo (USA). For PTT, BALB/c mice were subcutaneously injected with 5 × 10^5^ CT26 cells into the right flank and randomly sorted for treatment after 9 days when the tumor volumes reached approximately 100 mm^3^. PBS solution of SGNP or SGNP@PDA (50 µl, 50 fmol), or blank PBS (50 µl) were directly injected into the tumor followed by laser irradiation for 5 min (1 W/cm^2^) on the following day. The local tumor temperature was measured during laser irradiation by inserting a thermocouple probe into the tumor region. The photothermal reshaping of SGNP and SGNP@PDA in the tumors was analyzed by excising tumors 24 h after laser irradiation, followed by cutting the tissues into small pieces, and lysing cells using T-PER™ Tissue Protein Extraction Reagent mixed with Halt™ Protease Inhibitor Cocktails (Thermo Scientific). SGNP and SGNP@PDA were recovered by successive centrifugations and their morphological changes were examined using TEM. To study anti-tumor immunity induced by SGNP@PDA-mediated PTT, 50 µl PBS solution of SGNP@PDA (100 fmol) or blank PBS were directly injected into tumor followed by laser irradiation for 5 min (1 W/cm^2^) after 24 h. Blood was collected on day 7 and day 20 after laser irradiation for the analysis of PBMCs and immune serum, respectively. The immunological memory effect was investigated by re-challenging the survived mice with 5 × 10^5^ CT26 cells on the left flank after 55 days of the first tumor inoculation. The tumor growth was compared with naive mice that have not received sample injection and laser irradiation. For chemo-photothermal combination therapy in a bilateral tumor model, BALB/c mice were subcutaneously injected with 5 × 10^5^ CT26 cells into the right (primary tumor) and left (contralateral tumor) flank on days 0 and 6, respectively, and randomly sorted for treatment at day 9 when the primary tumor volumes reached approximately 100 mm^3^. The mice were injected intratumorally with 50 µl PBS solution of SGNP@PDA, DOX, SGNP@PDA + DOX with the doses at 100 fmol SGNP@PDA and 1.36 mg/kg DOX. Blank PBS was used as a control group. After 24 h, the primary tumors were exposed to laser irradiation for 5 min (1 W/cm^2^) along with temperature measurement by a thermocouple probe insertion. Tumor infiltrating lymphocytes were analyzed using flow cytometry for CD8+ T and NK cells after 7 days of laser irradiation. To measure the expression of HSP-70, tumors excised after 7 days of laser irradiation were cut into small pieces and lysed using T-PER™ Tissue Protein Extraction Reagent mixed with Halt™ Protease Inhibitor Cocktails. The lysis suspension was centrifuged to remove connective tissues and cell debris, and the concentration of HSP-70 was measured with ELISA by following manufacturer’s protocol (R&D Systems). To determine intratumoral DOX accumulation by SGNP@PDA + DOX treatment, both primary and contralateral tumors were excised after 24 h of sample administration and total tumor DOX levels were determined by acidified isopropanol extraction of whole tumor homogenates^57^. The tumor accumulation level was compared with the intravenously injected DOX to simulate translocation of DOX from primary to contralateral tumors after systemic circulation. The survived mice were re-challenged with 5 × 10^5^ CT26 cells on the left flank after 55 days of the first tumor inoculation to examine immunological memory effect. The sizes of the tumors were measured twice a week using a digital caliper, and the tumor volume was estimated by ellipsoidal calculation as *V* = (width)^2^ × length × π/6. The mice were euthanized when the tumors reached the maximum permitted size (1.5 cm in any dimension) or ulcerations occurred.

For chemo-photothermal combination therapy in the TC-1 submucosa-lung tumor model, C57BL/6 mice were injected with 5 × 10^4^ TC-1/luc cells (a gift from Dr. T.C. Wu) at a submucosal site of the inner lip on day 0, followed by intravenous injection of 1 × 10^5^ TC-1/luc cells on day 3. Animals were randomly sorted for treatment on day 10 when the lip tumor volumes reached approximately 50 mm^3^. The lip tumors were administered with 50 µl of PBS, SGNP@PDA, DOX, or SGNP@PDA + DOX with 100 fmol SGNP@PDA and/or 1.36 mg/kg DOX. On the following day, the lip tumors were exposed to laser irradiation for 5 min (1 W/cm^2^). In vivo bioluminescence imaging was performed using an IVIS optical imaging system (Caliper Life Sciences) and the signals were analyzed by the Living Image software. The animal study protocol was reviewed and approved by the Institutional Animal Care and Use Committee at the University of Michigan.

### Flow cytometric analysis

For analysis of tumor antigen-specific CD8+ T cells in systemic circulation, submandibular bleeding was performed after 7 days of laser irradiation and PBMCs were collected by removing red blood cells using ACK lysis buffer. PBMCs were then stained with AH1 peptide-MHC tetramer tagged with PE (H-2L^d^-restricted SPSYVYHQF, the NIH Tetramer Core Facility) and anti-CD8-APC (BD Biosciences, #553035). The generation of serum IgG specific to CT26 cells was analyzed by incubating CT26 cells with immune serum obtained at day 20 after laser irradiation, followed by anti-IgG-PE (eBioscience, #12-4010-87) secondary antibody staining. For tumor infiltrating cytotoxic lymphocytes analysis, tumor tissues were harvested on day 7 after laser irradiation, cut into small pieces, and treated with 1 mg/ml of collagenase type IV and 0.1 mg/ml of DNase I in RPMI for 30 min at 37 °C with gentle shaking. The cell suspensions were filtered through a 70-μm strainer, washed with FACS buffer (1% BSA in PBS), and then CD8 and NK cells were stained with the following antibody-fluorophore conjugates; CD8-APC and AH1 peptide-MHC tetramer-PE for CD8 cells, and CD3-PE (eBioscience, #12-0031-83), CD49b-APC (eBioscience, #17-5971-82), CD107a-PECy7 (BD Bioscience, #560647) for NK cells. For the analysis of surface expression of NKG2D ligand, the tumor cell suspension was stained with hamster anti-mouse MULT-1 (eBioscience, #14-5863-81) followed by secondary staining with goat anti-hamster IgG-PE (eBioscience, #12-4112-83). For the analysis of DC activation in tumor-draining lymph node, inguinal lymph nodes were harvested after 2 days of laser irradiation and treated with 1 mg/ml of collagenase type IV and 0.1 mg/ml of DNase I in RPMI for 30 min at 37 °C with gentle shaking. Lymph nodes were ground with the rubber end of a syringe, filtered through a 70-μm strainer, and washed with FACS buffer. DCs were stained with the following antibody-fluorophore conjugates; MHCII-PerCP-Cy5.5 (BD Bioscience, #562363), CD40-APC (eBioscience, #17-0401-81), and CD11c-PECy7 (BD Biosciences, #558079). In all flow cytometric analyses, antibodies were used at 1:100 dilution, and only live and intact cells were analyzed by suspending cells in DAPI solution and gating out DAPI-positive populations.

### In vivo depletion and blocking experiments

Bilateral tumor models were established on BALB/c mice, and primary tumors were treated with SGNP@PDA + DOX followed by laser irradiation according to the chemo-photothermal combination therapy regimen described earlier. Lymphocyte depletion was achieved using antibodies against CD4 (Bioxcell, clone GK1.5, #BP0003-1), CD8 (Bioxcell, clone 2.43, #BP0061), NK (Wako chemicals USA, Inc, Anti Asialo GM1, #986-10001), and neutrophil (BioXcell, clone 1A8, #BP0075-1). Immunoglobulin G (IgG) 2b (BioXcell, clone LTF-2, #BP0090) was used as an isotype control. Antibodies (200 µg) were administered intraperitoneally on days −2, 0, 2, 6, 13, and 20 of sample injection.

### Statistical analysis

Sample sizes were chosen based on the preliminary data from pilot experiments and previously published results in the literature. For animal studies, the mice were randomized to match similar average volume of the primary tumors and all procedures were performed in a non-blinded fashion. Statistical analysis was performed with Prism 6.0 software (GraphPad Software) by an unpaired Student’s *t*-test, one-way or two-way ANOVA with Bonferroni multiple comparisons post-test. Statistical significance for survival curve was calculated by the log-rank test. Data were approximately normally distributed and variance was similar between the groups. Statistical significance is indicated as *P < 0.05, **P < 0.01, ***P < 0.001, and ****P < 0.0001.

### Data availability

The authors declare that data supporting the findings of this study are available within the article and its Supplementary Information files. All relevant data can be provided by the authors upon reasonable request.

## Electronic supplementary material

Supplementary Information
