# Supplementary material for: Chemo-photothermal therapy combination elicits anti-tumor immunity against advanced metastatic cancer
Source: Nat Commun. 2018 Mar 14;9:1074. doi: 10.1038/s41467-018-03473-9 (PMC5852008; doi:10.1038/s41467-018-03473-9)
Supplement: Supplementary file 1 — Supplementary Information [file 41467_2018_3473_MOESM1_ESM.pdf]

Supplementary Information

Chemo-photothermal therapy combination elicits potent anti-tumor immunity against advanced metastatic cancer

Jutaek Nam[1,2,#], Sejin Son[1,2,#], Lukasz J. Ochyl[1,2], Rui Kuai[1,2], Anna Schwendeman[1,2], and James J. Moon[1,2,3,*]

1. Department of Pharmaceutical Sciences, University of Michigan, Ann Arbor, MI 48109, USA

2. Biointerfaces Institute, University of Michigan, Ann Arbor, MI 48109, USA

3. Department of Biomedical Engineering, University of Michigan, Ann Arbor, MI 48109, USA

# These authors contributed equally to this work

Correspondence should be addressed to J.J.M. (moonjj@umich.edu)

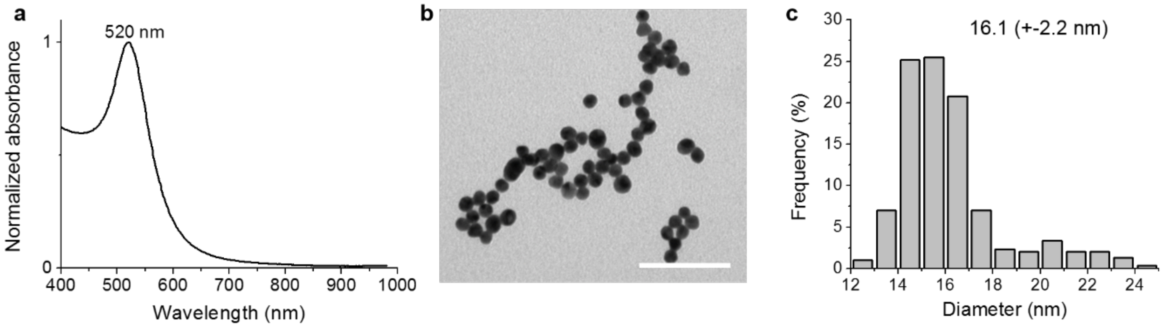

Supplementary Figure 1. Absorption spectrum (a), TEM image (b) and corresponding size distribution (c) of seed GNP. Scale bar is 100 nm in (b) and the number presents mean ± s.d. in (c).

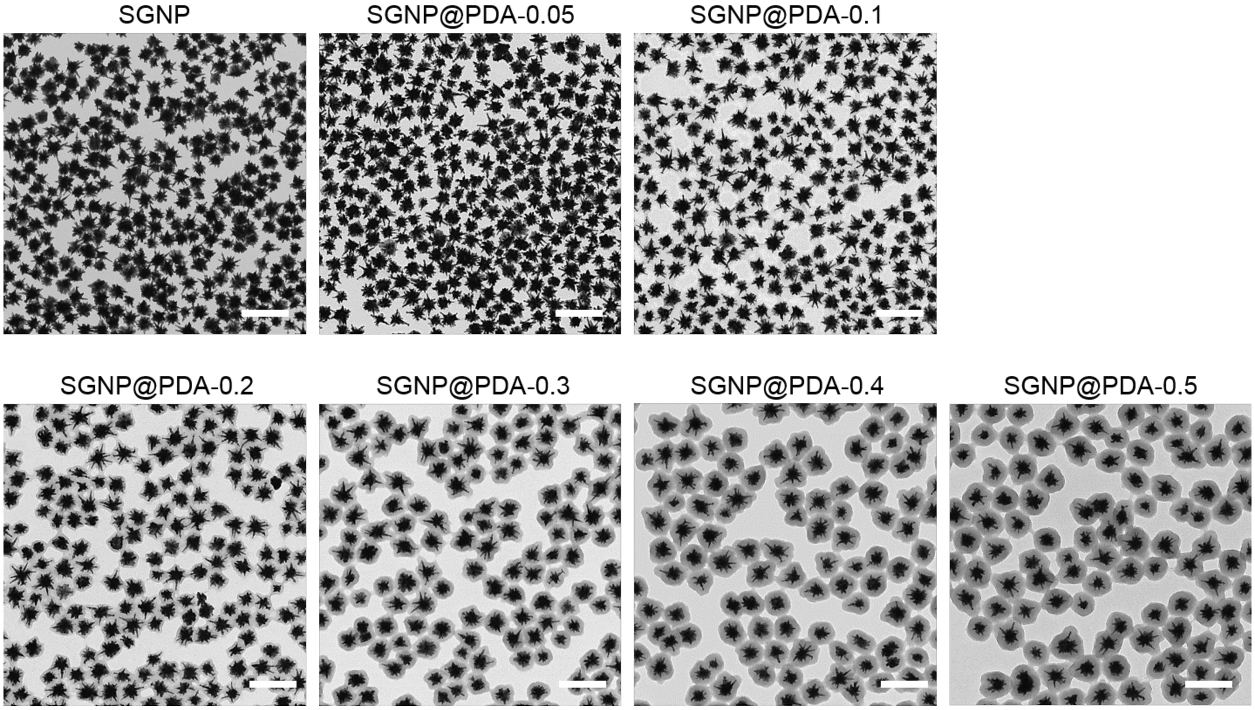

Supplementary Figure 2. Low magnification TEM images of SGNP and SGNP@PDAs. Scale bars are 200 nm.

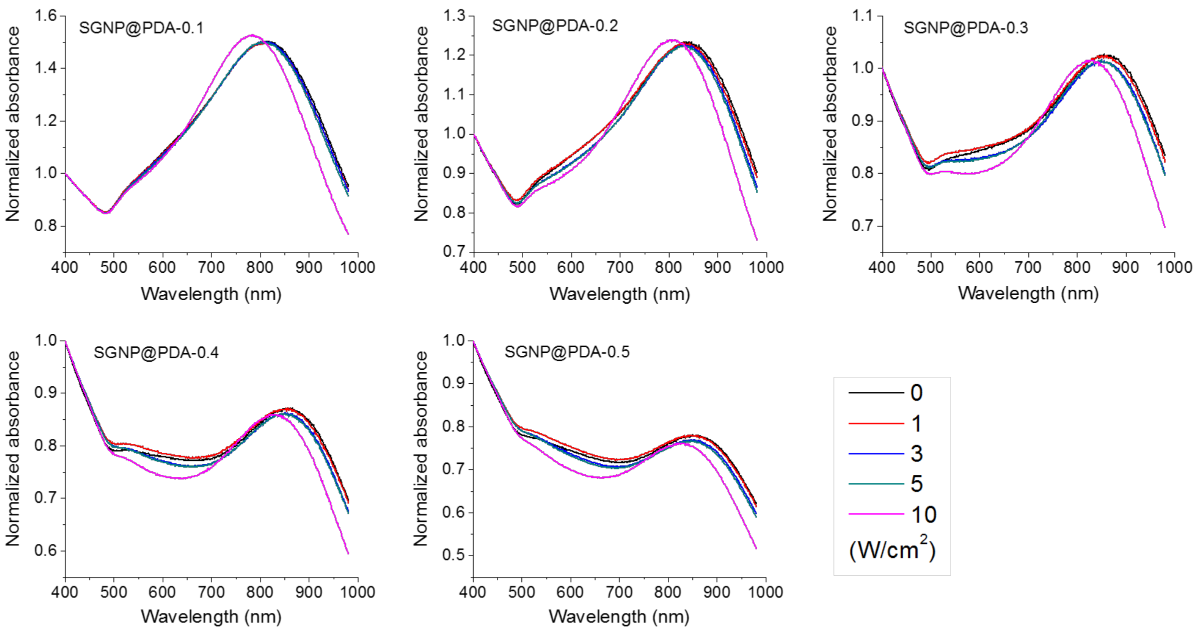

Supplementary Figure 3. Absorption spectra of SGNP@PDAs measured after laser irradiation at 0 (no irradiation), 1, 3, 5, 10 W/cm^2 for 30 min.

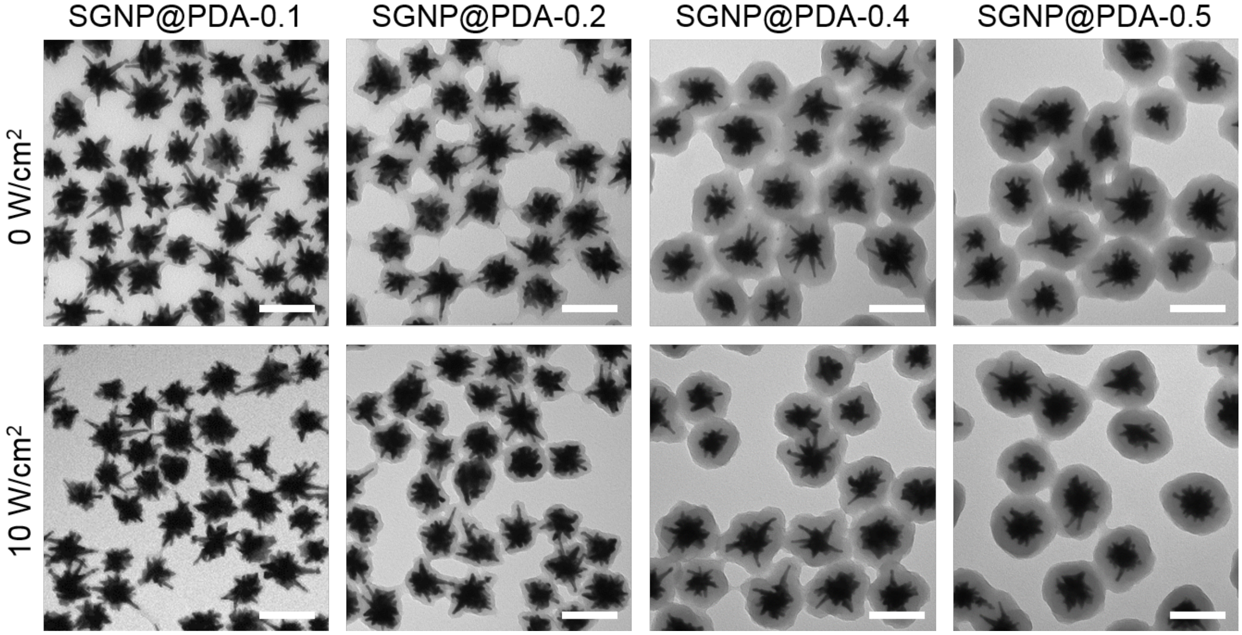

Supplementary Figure 4. TEM images of SGNP@PDAs before (0 W/cm^2) and after (10 W/cm^2) laser irradiation at 10 W/cm^2 for 30 min. Scale bars are 100 nm.

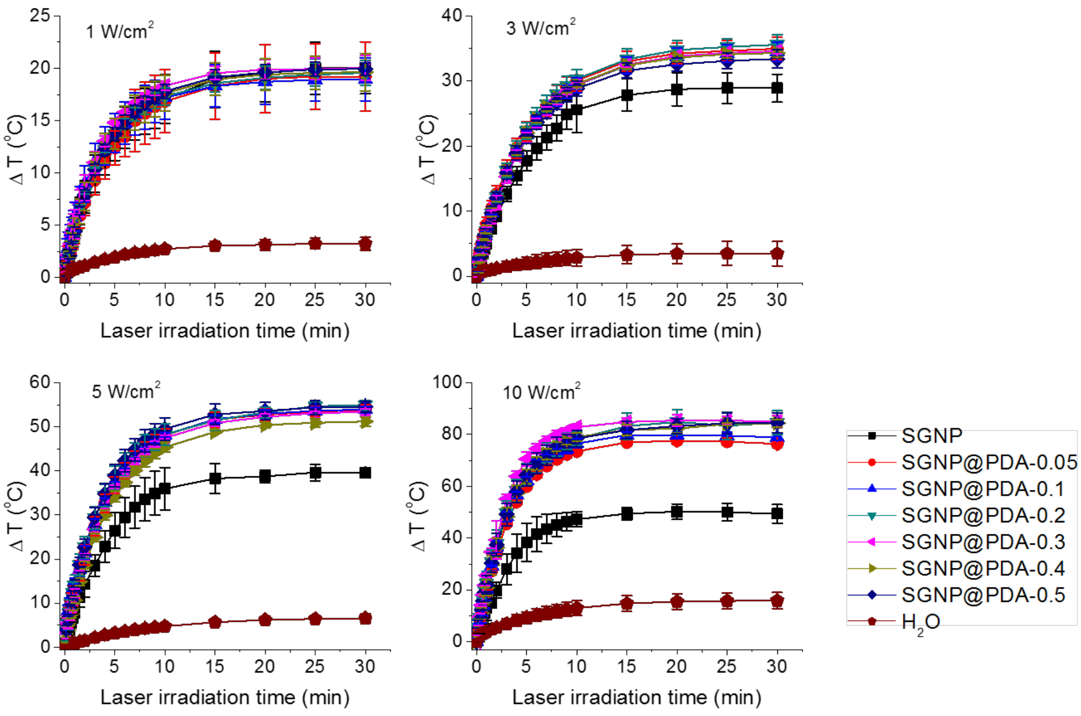

Supplementary Figure 5. Temperature increase of SGNP, SGNP@PDAs, and deionized water (d.i.water) measured during 30 min of laser irradiations at 1, 3, 5, 10 W/cm^2.

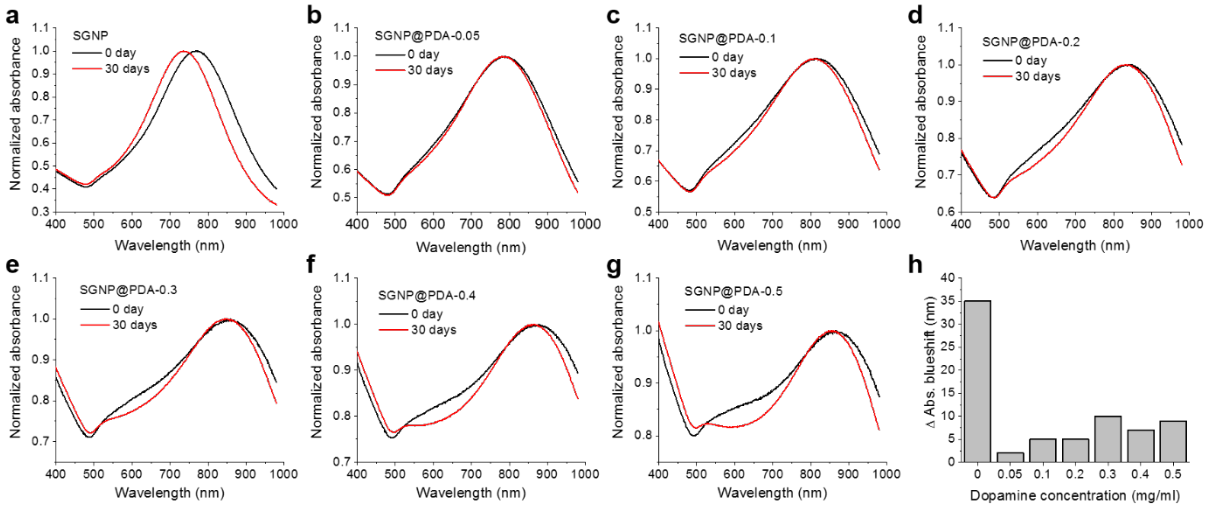

Supplementary Figure 6. Absorption spectra of SGNP and SGNP@PDAs before (0 day) and after (30 days) storage at 4 °C for 30 days (a-g) and corresponding blue-shift in their absorption peaks (h).

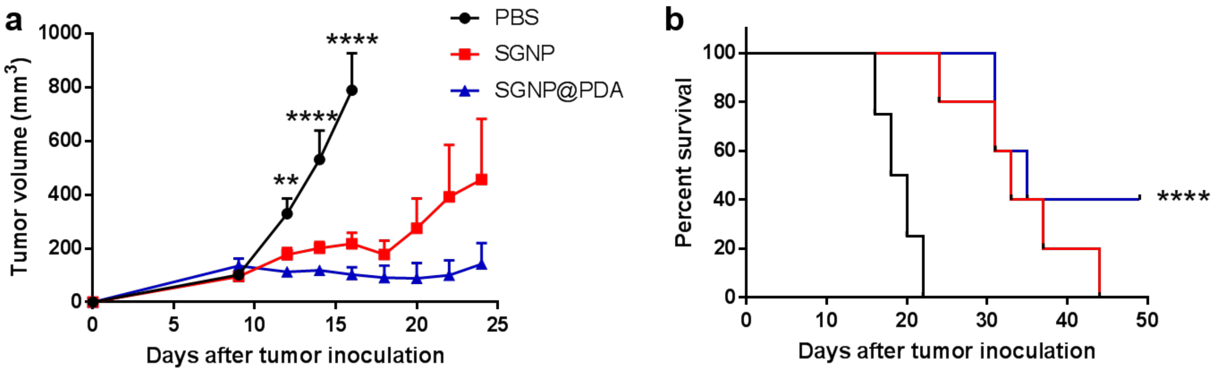

Supplementary Figure 7. Tumor growth (a) and Kaplan–Meier survival curve (b) of CT26 tumor-bearing mice after treatment of PBS, SGNP, or SGNP@PDA followed by laser irradiation. The data show mean ± s.d. (n = 5). ∗∗P < 0.01 and ∗∗∗∗P < 0.0001 between PBS and SGNP@PDA, analysed by two-way ANOVA with Bonferroni multiple comparisons post-test (a) or log-rank (Mantel–Cox) test (b).

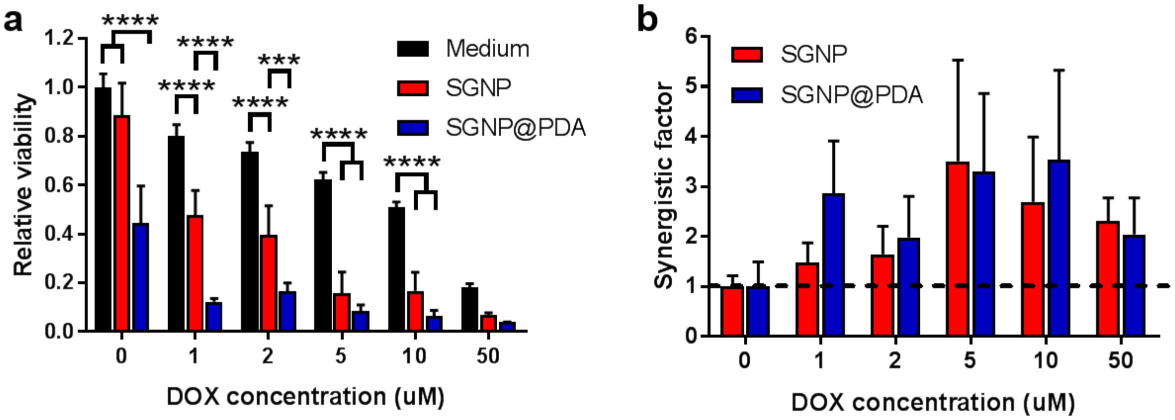

Supplementary Figure 8. (a) Viability of CT26 cells after co-treatment of varying concentration of DOX with 1 pM of SGNP or SGNP@PDA followed by laser irradiation at 10 W/cm^2 for 5 min and further 24 h incubation. “Medium” indicates DOX treatment alone. (b) Synergistic factor of combination therapy calculated based on the viability in (a). The data show mean ± s.d. (n = 3). ***$P < 0.001$, and ****$P < 0.0001$, analysed by two-way ANOVA with Bonferroni multiple comparisons post-test.

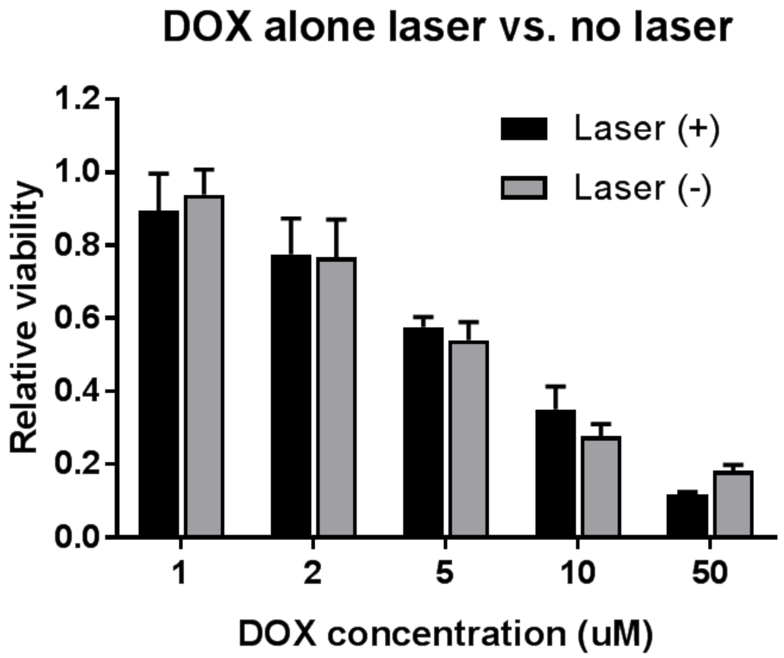

Supplementary Figure 9. Viability of CT26 cells treated with DOX (Laser-) or DOX followed by laser irradiation (Laser+). The data show mean ± s.d. (n = 3).

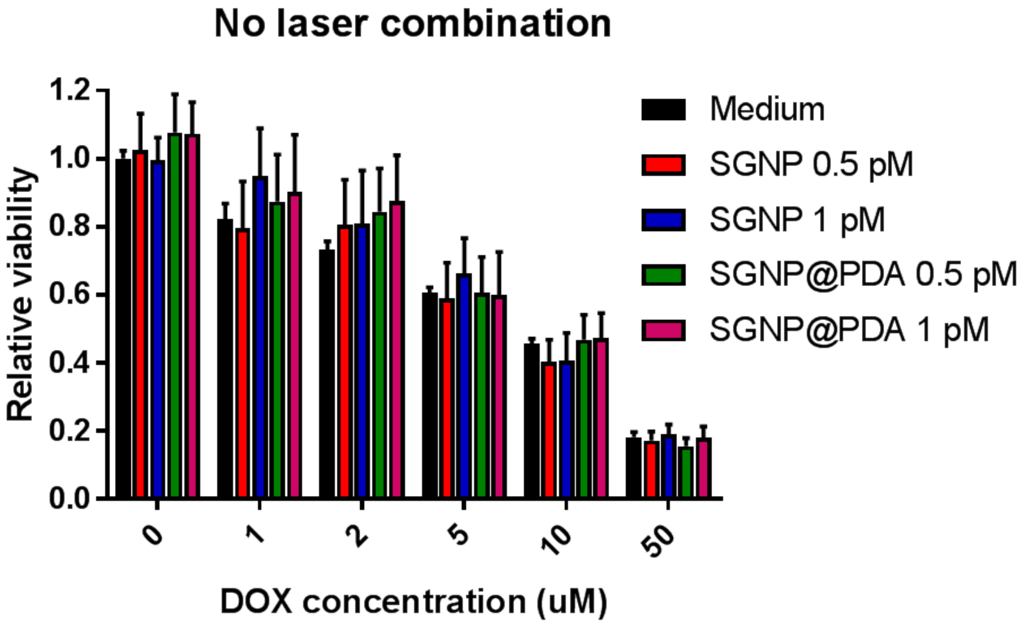

Supplementary Figure 10. Viability of CT26 cells after co-treatment of varying concentrations of DOX with 0.5 pM or 1 pM of SGNP or SGNP@PDA for 24 h in dark condition. The data show mean ± s.d. (n = 3).

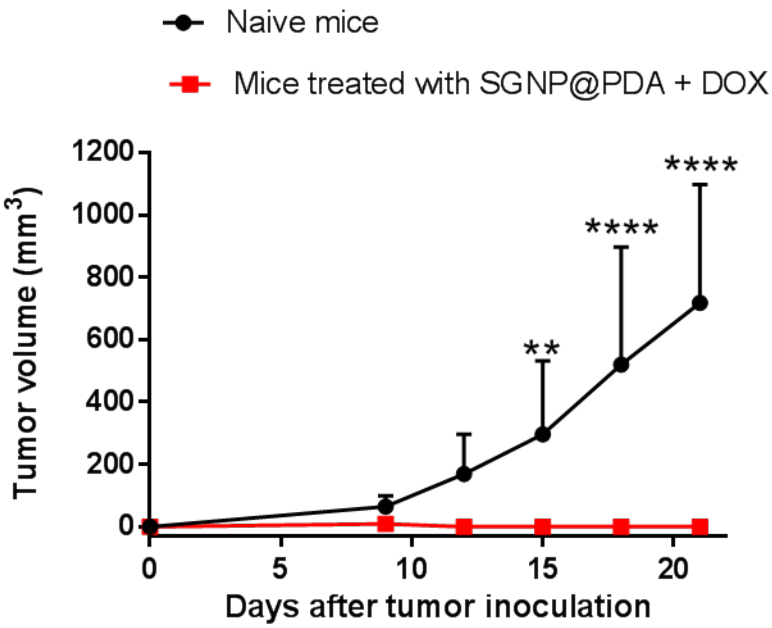

Supplementary Figure 11. The average growth curve of re-challenged tumors. The data show mean ± s.d. (n = 11-13). ***P < 0.001 and ****P < 0.0001, analysed by two-way ANOVA with Bonferroni multiple comparisons post-test.

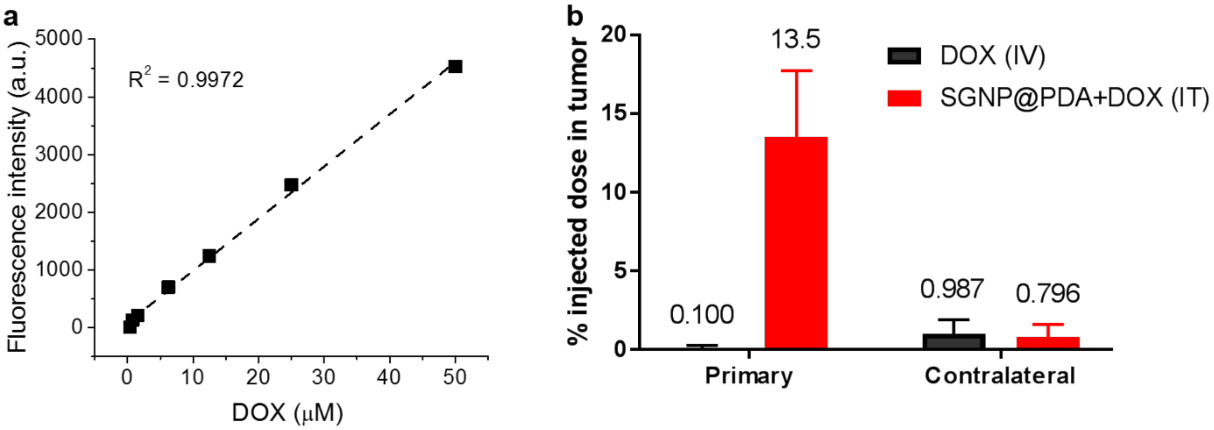

Supplementary Figure 12. (a) Standard curve of DOX concentration vs. fluorescence intensity measured with excitation and emission at 485 and 590 nm, respectively. (b) Tumor accumulation of DOX analysed by acidified isopropanol extraction of whole tumor homogenates at 24 h after intravenous injection of DOX or intratumoral injection of SGNP@PDA+DOX into the primary tumors. The DOX concentration was deduced from the standard curve in (a) by measuring fluorescence intensity. The data show mean ± s.d. (n = 4-5).

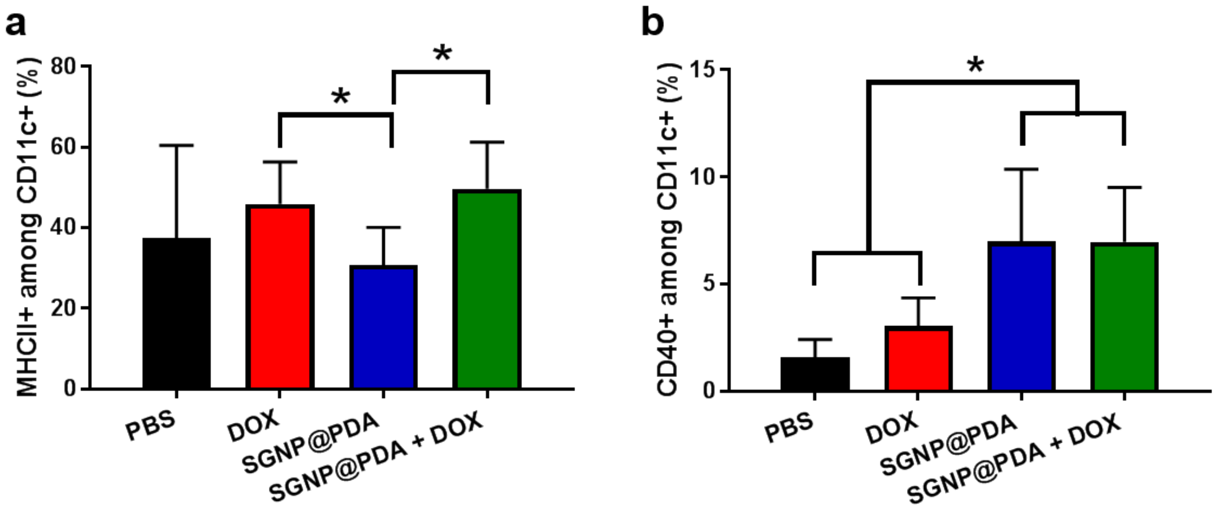

Supplementary Figure 13. Surface expression level of MHCII and CD40 among CD11c+ dendritic cells in tumor-draining lymph node analysed by flow cytometry. The data show mean ± s.d. (n = 5-10). $*P < 0.05$, analysed by one-way ANOVA with Bonferroni multiple comparisons post-test.
